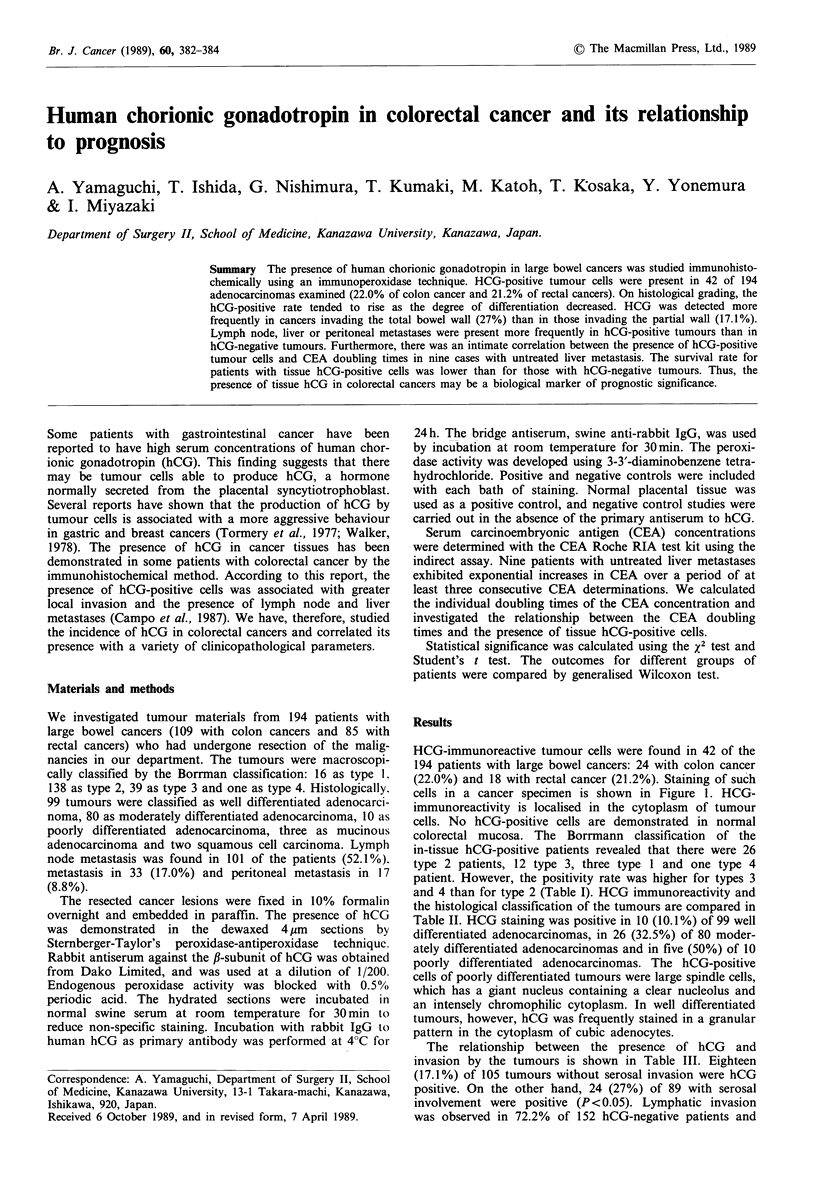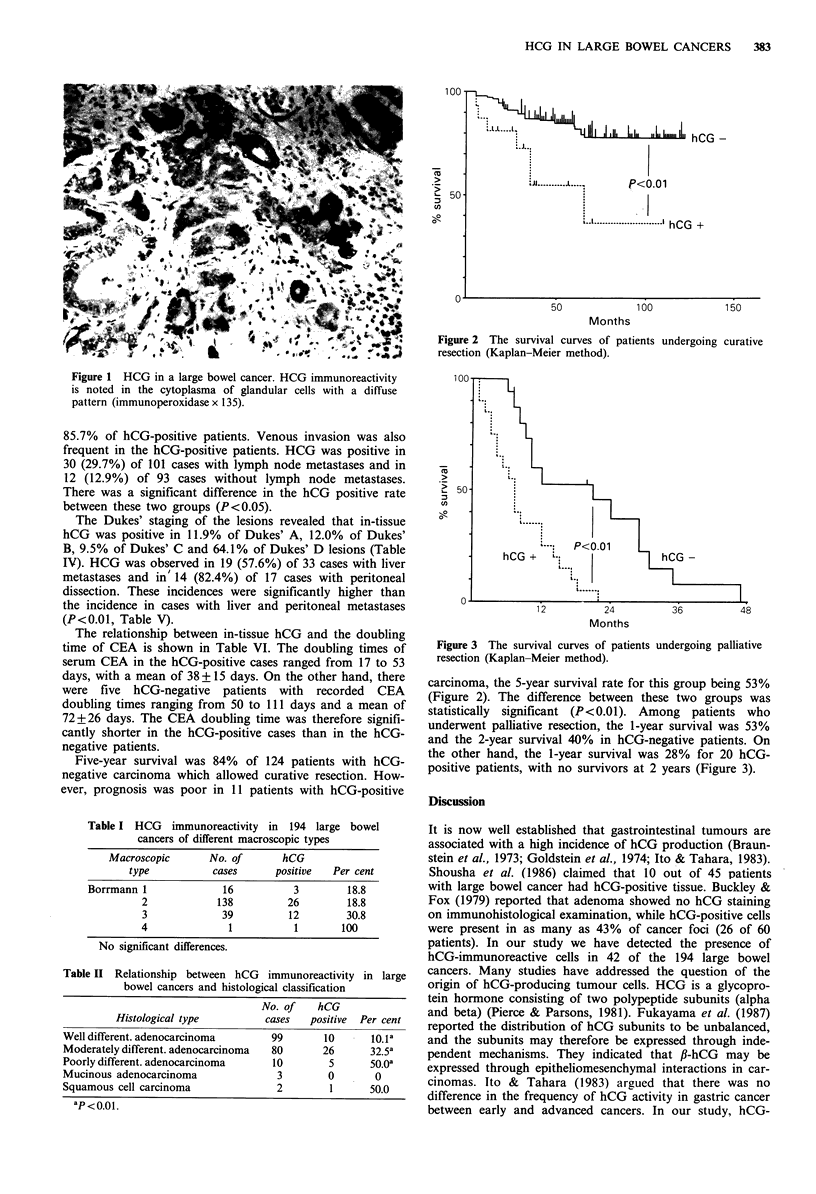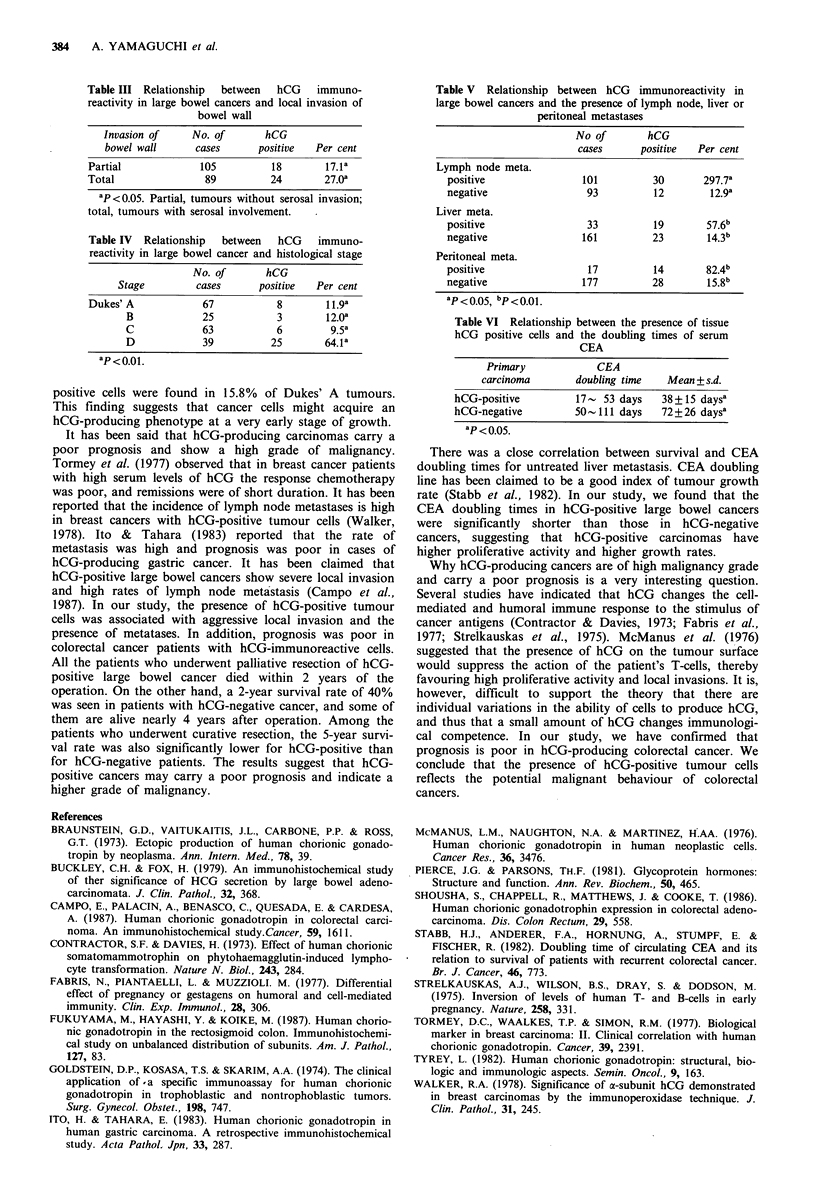# Human chorionic gonadotropin in colorectal cancer and its relationship to prognosis.

**DOI:** 10.1038/bjc.1989.289

**Published:** 1989-09

**Authors:** A. Yamaguchi, T. Ishida, G. Nishimura, T. Kumaki, M. Katoh, T. Kosaka, Y. Yonemura, I. Miyazaki

**Affiliations:** Department of Surgery II, School of Medicine, Kanazawa University, Japan.

## Abstract

**Images:**


Br. J. Cancer (1989), 60, 382–384 © The Macmillan Press, Ltd., 1989

### Human chorionic gonadotropin in colorectal cancer and its relationship to prognosis

A. Yamaguchi, T. Ishida, G. Nishimura, T. Kumaki, M. Katoh, T. Kosaka, Y. Yonemura & I. Miyazaki

*Department of Surgery II, School of Medicine, Kanazawa University, Kanazawa, Japan.*

**Summary** The presence of human chorionic gonadotropin in large bowel cancers was studied immunohistochemically using an immunoperoxidase technique. HCG-positive tumour cells were present in 42 of 194 adenocarcinomas examined (22.0% of colon cancer and 21.2% of rectal cancers). On histological grading, the hCG-positive rate tended to rise as the degree of differentiation decreased. HCG was detected more frequently in cancers invading the total bowel wall (27%) than in those invading the partial wall (17.1%). Lymph node, liver or peritoneal metastases were present more frequently in hCG-positive tumours than in hCG-negative tumours. Furthermore, there was an intimate correlation between the presence of hCG-positive tumour cells and CEA doubling times in nine cases with untreated liver metastasis. The survival rate for patients with tissue hCG-positive cells was lower than for those with hCG-negative tumours. Thus, the presence of tissue hCG in colorectal cancers may be a biological marker of prognostic significance.

Some patients with gastrointestinal cancer have been reported to have high serum concentrations of human chorionic gonadotropin (hCG). This finding suggests that there may be tumour cells able to produce hCG, a hormone normally secreted from the placental syncytiotrophoblast. Several reports have shown that the production of hCG by tumour cells is associated with a more aggressive behaviour in gastric and breast cancers (Tormery *et al.*, 1977; Walker, 1978). The presence of hCG in cancer tissues has been demonstrated in some patients with colorectal cancer by the immunohistochemical method. According to this report, the presence of hCG-positive cells was associated with greater local invasion and the presence of lymph node and liver metastases (Campo *et al.*, 1987). We have, therefore, studied the incidence of hCG in colorectal cancers and correlated its presence with a variety of clinicopathological parameters.

### Materials and methods

We investigated tumour materials from 194 patients with large bowel cancers (109 with colon cancers and 85 with rectal cancers) who had undergone resection of the malignancies in our department. The tumours were macroscopically classified by the Borrman classification: 16 as type 1, 138 as type 2, 39 as type 3 and one as type 4. Histologically, 99 tumours were classified as well differentiated adenocarcinoma, 80 as moderately differentiated adenocarcinoma, 10 as poorly differentiated adenocarcinoma, three as mucinous adenocarcinoma and two squamous cell carcinoma. Lymph node metastasis was found in 101 of the patients (52.1%), metastasis in 33 (17.0%) and peritoneal metastasis in 17 (8.8%).

The resected cancer lesions were fixed in 10% formalin overnight and embedded in paraffin. The presence of hCG was demonstrated in the dewaxed 4 $\mu$m sections by Sternberger-Taylor's peroxidase-antiperoxidase technique. Rabbit antiserum against the $\beta$-subunit of hCG was obtained from Dako Limited, and was used at a dilution of 1/200. Endogenous peroxidase activity was blocked with 0.5% periodic acid. The hydrated sections were incubated in normal swine serum at room temperature for 30 min to reduce non-specific staining. Incubation with rabbit IgG to human hCG as primary antibody was performed at 4°C for 24 h. The bridge antiserum, swine anti-rabbit IgG, was used by incubation at room temperature for 30 min. The peroxidase activity was developed using 3-3′-diaminobenzene tetrahydrochloride. Positive and negative controls were included with each bath of staining. Normal placental tissue was used as a positive control, and negative control studies were carried out in the absence of the primary antiserum to hCG.

Serum carcinoembryonic antigen (CEA) concentrations were determined with the CEA Roche RIA test kit using the indirect assay. Nine patients with untreated liver metastases exhibited exponential increases in CEA over a period of at least three consecutive CEA determinations. We calculated the individual doubling times of the CEA concentration and investigated the relationship between the CEA doubling times and the presence of tissue hCG-positive cells.

Statistical significance was calculated using the $\chi^2$ test and Student's $t$ test. The outcomes for different groups of patients were compared by generalised Wilcoxon test.

### Results

HCG-immunoreactive tumour cells were found in 42 of the 194 patients with large bowel cancers: 24 with colon cancer (22.0%) and 18 with rectal cancer (21.2%). Staining of such cells in a cancer specimen is shown in Figure 1. HCG-immunoreactivity is localised in the cytoplasm of tumour cells. No hCG-positive cells are demonstrated in normal colorectal mucosa. The Borrmann classification of the in-tissue hCG-positive patients revealed that there were 26 type 2 patients, 12 type 3, three type 1 and one type 4 patient. However, the positivity rate was higher for types 3 and 4 than for type 2 (Table I). HCG immunoreactivity and the histological classification of the tumours are compared in Table II. HCG staining was positive in 10 (10.1%) of 99 well differentiated adenocarcinomas, in 26 (32.5%) of 80 moderately differentiated adenocarcinomas and in five (50%) of 10 poorly differentiated adenocarcinomas. The hCG-positive cells of poorly differentiated tumours were large spindle cells, which has a giant nucleus containing a clear nucleolus and an intensely chromophilic cytoplasm. In well differentiated tumours, however, hCG was frequently stained in a granular pattern in the cytoplasm of cubic adenocytes.

The relationship between the presence of hCG and invasion by the tumours is shown in Table III. Eighteen (17.1%) of 105 tumours without serosal invasion were hCG positive. On the other hand, 24 (27%) of 89 with serosal involvement were positive ($P<0.05$). Lymphatic invasion was observed in 72.2% of 152 hCG-negative patients and

Correspondence: A. Yamaguchi, Department of Surgery II, School of Medicine, Kanazawa University, 13-1 Takara-machi, Kanazawa, Ishikawa, 920, Japan.
Received 6 October 1989, and in revised form, 7 April 1989.

**Figure 1** HCG in a large bowel cancer. HCG immunoreactivity is noted in the cytoplasma of glandular cells with a diffuse pattern (immunoperoxidase x 135).

85.7% of hCG-positive patients. Venous invasion was also frequent in the hCG-positive patients. HCG was positive in 30 (29.7%) of 101 cases with lymph node metastases and in 12 (12.9%) of 93 cases without lymph node metastases. There was a significant difference in the hCG positive rate between these two groups ($P<0.05$).

The Dukes' staging of the lesions revealed that in-tissue hCG was positive in 11.9% of Dukes' A, 12.0% of Dukes' B, 9.5% of Dukes' C and 64.1% of Dukes' D lesions (Table IV). HCG was observed in 19 (57.6%) of 33 cases with liver metastases and in 14 (82.4%) of 17 cases with peritoneal dissection. These incidences were significantly higher than the incidence in cases with liver and peritoneal metastases ($P<0.01$, Table V).

The relationship between in-tissue hCG and the doubling time of CEA is shown in Table VI. The doubling times of serum CEA in the hCG-positive cases ranged from 17 to 53 days, with a mean of $38 \pm 15$ days. On the other hand, there were five hCG-negative patients with recorded CEA doubling times ranging from 50 to 111 days and a mean of $72 \pm 26$ days. The CEA doubling time was therefore significantly shorter in the hCG-positive cases than in the hCG-negative patients.

Five-year survival was 84% of 124 patients with hCG-negative carcinoma which allowed curative resection. However, prognosis was poor in 11 patients with hCG-positive

**Table I** HCG immunoreactivity in 194 large bowel cancers of different macroscopic types

| *Macroscopic type* | *No. of cases* | *hCG positive* | *Per cent* |
|---|---|---|---|
| Borrmann 1 | 16 | 3 | 18.8 |
| 2 | 138 | 26 | 18.8 |
| 3 | 39 | 12 | 30.8 |
| 4 | 1 | 1 | 100 |

No significant differences.

**Table II** Relationship between hCG immunoreactivity in large bowel cancers and histological classification

| *Histological type* | *No. of cases* | *hCG positive* | *Per cent* |
|---|---|---|---|
| Well different. adenocarcinoma | 99 | 10 | 10.1[a] |
| Moderately different. adenocarcinoma | 80 | 26 | 32.5[a] |
| Poorly different. adenocarcinoma | 10 | 5 | 50.0[a] |
| Mucinous adenocarcinoma | 3 | 0 | 0 |
| Squamous cell carcinoma | 2 | 1 | 50.0 |

[a] $P<0.01$.

**Figure 2** The survival curves of patients undergoing curative resection (Kaplan–Meier method).

**Figure 3** The survival curves of patients undergoing palliative resection (Kaplan–Meier method).

carcinoma, the 5-year survival rate for this group being 53% (Figure 2). The difference between these two groups was statistically significant ($P<0.01$). Among patients who underwent palliative resection, the 1-year survival was 53% and the 2-year survival 40% in hCG-negative patients. On the other hand, the 1-year survival was 28% for 20 hCG-positive patients, with no survivors at 2 years (Figure 3).

### Discussion

It is now well established that gastrointestinal tumours are associated with a high incidence of hCG production (Braunstein *et al.*, 1973; Goldstein *et al.*, 1974; Ito & Tahara, 1983). Shousha *et al.* (1986) claimed that 10 out of 45 patients with large bowel cancer had hCG-positive tissue. Buckley & Fox (1979) reported that adenoma showed no hCG staining on immunohistological examination, while hCG-positive cells were present in as many as 43% of cancer foci (26 of 60 patients). In our study we have detected the presence of hCG-immunoreactive cells in 42 of the 194 large bowel cancers. Many studies have addressed the question of the origin of hCG-producing tumour cells. HCG is a glycoprotein hormone consisting of two polypeptide subunits (alpha and beta) (Pierce & Parsons, 1981). Fukayama *et al.* (1987) reported the distribution of hCG subunits to be unbalanced, and the subunits may therefore be expressed through independent mechanisms. They indicated that $\beta$-hCG may be expressed through epitheliomesenchymal interactions in carcinomas. Ito & Tahara (1983) argued that there was no difference in the frequency of hCG activity in gastric cancer between early and advanced cancers. In our study, hCG-

**Table III** Relationship between hCG immunoreactivity in large bowel cancers and local invasion of bowel wall

| *Invasion of bowel wall* | *No. of cases* | *hCG positive* | *Per cent* |
|---|---|---|---|
| Partial | 105 | 18 | 17.1[a] |
| Total | 89 | 24 | 27.0[a] |

[a]$P<0.05$. Partial, tumours without serosal invasion; total, tumours with serosal involvement.

**Table IV** Relationship between hCG immunoreactivity in large bowel cancer and histological stage

| *Stage* | *No. of cases* | *hCG positive* | *Per cent* |
|---|---|---|---|
| Dukes' A | 67 | 8 | 11.9[a] |
| B | 25 | 3 | 12.0[a] |
| C | 63 | 6 | 9.5[a] |
| D | 39 | 25 | 64.1[a] |

[a]$P<0.01$.

positive cells were found in 15.8% of Dukes' A tumours. This finding suggests that cancer cells might acquire an hCG-producing phenotype at a very early stage of growth.

It has been said that hCG-producing carcinomas carry a poor prognosis and show a high grade of malignancy. Tormey *et al.* (1977) observed that in breast cancer patients with high serum levels of hCG the response chemotherapy was poor, and remissions were of short duration. It has been reported that the incidence of lymph node metastases is high in breast cancers with hCG-positive tumour cells (Walker, 1978). Ito & Tahara (1983) reported that the rate of metastasis was high and prognosis was poor in cases of hCG-producing gastric cancer. It has been claimed that hCG-positive large bowel cancers show severe local invasion and high rates of lymph node metastasis (Campo *et al.*, 1987). In our study, the presence of hCG-positive tumour cells was associated with aggressive local invasion and the presence of metatases. In addition, prognosis was poor in colorectal cancer patients with hCG-immunoreactive cells. All the patients who underwent palliative resection of hCG-positive large bowel cancer died within 2 years of the operation. On the other hand, a 2-year survival rate of 40% was seen in patients with hCG-negative cancer, and some of them are alive nearly 4 years after operation. Among the patients who underwent curative resection, the 5-year survival rate was also significantly lower for hCG-positive than for hCG-negative patients. The results suggest that hCG-positive cancers may carry a poor prognosis and indicate a higher grade of malignancy.

**Table V** Relationship between hCG immunoreactivity in large bowel cancers and the presence of lymph node, liver or peritoneal metastases

| | *No of cases* | *hCG positive* | *Per cent* |
|---|---|---|---|
| Lymph node meta. | | | |
| positive | 101 | 30 | 297.7[a] |
| negative | 93 | 12 | 12.9[a] |
| Liver meta. | | | |
| positive | 33 | 19 | 57.6[b] |
| negative | 161 | 23 | 14.3[b] |
| Peritoneal meta. | | | |
| positive | 17 | 14 | 82.4[b] |
| negative | 177 | 28 | 15.8[b] |

[a]$P<0.05$, [b]$P<0.01$.

**Table VI** Relationship between the presence of tissue hCG positive cells and the doubling times of serum CEA

| *Primary carcinoma* | *CEA doubling time* | *Mean ± s.d.* |
|---|---|---|
| hCG-positive | 17∼ 53 days | 38±15 days[a] |
| hCG-negative | 50∼111 days | 72±26 days[a] |

[a]$P<0.05$.

There was a close correlation between survival and CEA doubling times for untreated liver metastasis. CEA doubling line has been claimed to be a good index of tumour growth rate (Stabb *et al.*, 1982). In our study, we found that the CEA doubling times in hCG-positive large bowel cancers were significantly shorter than those in hCG-negative cancers, suggesting that hCG-positive carcinomas have higher proliferative activity and higher growth rates.

Why hCG-producing cancers are of high malignancy grade and carry a poor prognosis is a very interesting question. Several studies have indicated that hCG changes the cell-mediated and humoral immune response to the stimulus of cancer antigens (Contractor & Davies, 1973; Fabris *et al.*, 1977; Strelkauskas *et al.*, 1975). McManus *et al.* (1976) suggested that the presence of hCG on the tumour surface would suppress the action of the patient's T-cells, thereby favouring high proliferative activity and local invasions. It is, however, difficult to support the theory that there are individual variations in the ability of cells to produce hCG, and thus that a small amount of hCG changes immunological competence. In our study, we have confirmed that prognosis is poor in hCG-producing colorectal cancer. We conclude that the presence of hCG-positive tumour cells reflects the potential malignant behaviour of colorectal cancers.

### References

BRAUNSTEIN, G.D., VAITUKAITIS, J.L., CARBONE, P.P. & ROSS, G.T. (1973). Ectopic production of human chorionic gonadotropin by neoplasma. *Ann. Intern. Med.*, **78**, 39.

BUCKLEY, C.H. & FOX, H. (1979). An immunohistochemical study of ther significance of HCG secretion by large bowel adenocarcinomata. *J. Clin. Pathol.*, **32**, 368.

CAMPO, E., PALACIN, A., BENASCO, C., QUESADA, E. & CARDESA, A. (1987). Human chorionic gonadotropin in colorectal carcinoma. An immunohistochemical study.*Cancer*, **59**, 1611.

CONTRACTOR, S.F. & DAVIES, H. (1973). Effect of human chorionic somatomammotrophin on phytohaemagglutin-induced lymphocyte transformation. *Nature N. Biol.*, **243**, 284.

FABRIS, N., PIANTAELLI, L. & MUZZIOLI. M. (1977). Differential effect of pregnancy or gestagens on humoral and cell-mediated immunity. *Clin. Exp. Immunol.*, **28**, 306.

FUKUYAMA, M., HAYASHI, Y. & KOIKE, M. (1987). Human chorionic gonadotropin in the rectosigmoid colon. Immunohistochemical study on unbalanced distribution of subunits. *Am. J. Pathol.*, **127**, 83.

GOLDSTEIN, D.P., KOSASA, T.S. & SKARIM, A.A. (1974). The clinical application of a specific immunoassay for human chorionic gonadotropin in trophoblastic and nontrophoblastic tumors. *Surg. Gynecol. Obstet.*, **198**, 747.

ITO, H. & TAHARA, E. (1983). Human chorionic gonadotropin in human gastric carcinoma. A retrospective immunohistochemical study. *Acta Pathol. Jpn*, **33**, 287.

McMANUS, L.M., NAUGHTON, N.A. & MARTINEZ, H.AA. (1976). Human chorionic gonadotropin in human neoplastic cells. *Cancer Res.*, **36**, 3476.

PIERCE, J.G. & PARSONS, TH.F. (1981). Glycoprotein hormones: Structure and function. *Ann. Rev. Biochem.*, **50**, 465.

SHOUSHA, S., CHAPPELL, R., MATTHEWS, J. & COOKE, T. (1986). Human chorionic gonadotrophin expression in colorectal adenocarcinoma. *Dis. Colon Rectum*, **29**, 558.

STABB, H.J., ANDERER, F.A., HORNUNG, A., STUMPF, E. & FISCHER, R. (1982). Doubling time of circulating CEA and its relation to survival of patients with recurrent colorectal cancer. *Br. J. Cancer*, **46**, 773.

STRELKAUSKAS, A.J., WILSON, B.S., DRAY, S. & DODSON, M. (1975). Inversion of levels of human T- and B-cells in early pregnancy. *Nature*, **258**, 331.

TORMEY, D.C., WAALKES, T.P. & SIMON, R.M. (1977). Biological marker in breast carcinoma: II. Clinical correlation with human chorionic gonadotropin. *Cancer*, **39**, 2391.

TYREY, L. (1982). Human chorionic gonadotropin: structural, biologic and immunologic aspects. *Semin. Oncol.*, **9**, 163.

WALKER, R.A. (1978). Significance of α-subunit hCG demonstrated in breast carcinomas by the immunoperoxidase technique. *J. Clin. Pathol.*, **31**, 245.